# Prognosis and Histological Classification in Elderly Patients with ANCA-Glomerulonephritis: A Registry-Based Cohort Study

**DOI:** 10.1155/2018/7581567

**Published:** 2018-05-31

**Authors:** Rune Bjørneklett, Leif Bostad, Anne-Siri Fismen

**Affiliations:** ^1^Department of Clinical Medicine, University of Bergen, Jonas Lies vei 91b, 5021 Bergen, Norway; ^2^Emergency Care Clinic, Haukeland University Hospital, Jonas Lies vei 65, 5021 Bergen, Norway; ^3^Department of Pathology, Haukeland University Hospital, Jonas Lies vei 65, 5021 Bergen, Norway; ^4^Faculty of Health and Social Sciences, Western Norway University of Applied Sciences, Inndalsveien 28, 5063 Bergen, Norway

## Abstract

**Background:**

The value of a histologic classification scheme to classify patients with anti-neutrophil cytoplasmic antibody-associated glomerulonephritis (ANCA-GN) into focal, mixed, crescentic, and sclerotic types for predicting risk of end-stage renal disease (ESRD) is well documented. However, the prognostic value of histological classification specifically in elderly patients (≥70 years) with ANCA-GN has not previously been investigated.

**Methods:**

Patients with biopsy-verified pauci-immune necrotizing glomerulonephritis were identified from the Norwegian Kidney Biopsy Registry between 1991 and 2012 and those ≥70 years of age at the time of diagnosis and having positive anti-neutrophil cytoplasmic antibody serology were included in this study. The incidence rate of ESRD and/or death was determined by linking the study cohort to the Norwegian Renal Registry and the Population Registry of Norway. The ESRD-free survival and patient survival were compared between the 4 histological types.

**Results:**

Of the 81 patients included, 20 progressed to ESRD and 34 died. The 1-year and 5-year ESRD-free survival varied between histological groups (*p* = 0.003) as follows: focal, 97% and 97%, respectively; mixed, 70% and 57%; crescentic, 76% and 63%; and sclerotic, 49% and 49%. Patient survival did not differ significantly between groups (*p* = 0.30).

**Conclusion:**

Histological classification in elderly patients with ANCA-GN is useful for predicting ESRD but not survival.

## 1. Introduction

Anti-neutrophil cytoplasmic antibody (ANCA) associated vasculitis (AAV), which affects multiple organs, has historically been associated with a high risk of long-term morbidity and mortality. The introduction of cyclophosphamide treatment, more than 5 decades ago, has greatly improved the prognosis of these patients [1]. However, even with modern treatment, the risk of AAV-associated morbidity and mortality remains high [2–4]. Although AAV can occur at any age, the risk of a fatal course of AAV is particularly high among elderly patients [5, 6], with the risk of death at 1 year being close to 50% among patients aged >80 years [7]. Kidney involvement, in the form of focal necrotizing glomerulonephritis (ANCA-GN), is frequent in patients with AAV and is associated with a poorer clinical outcome, including a substantial risk for end-stage renal disease (ESRD) necessitating chronic renal replacement therapy, in the form of dialysis or kidney transplantation [4]. Diagnostic kidney biopsy plays an important role in establishing and confirming the diagnosis of ANCA-GN [8, 9], as well as conveying important prognostic information [10–18]. The most frequently used and validated model to assess the prognostic information from kidney biopsies for ANCA-GN diagnosis is a histological classification scheme that classifies patients into 4 types:** focal** (*≥50*%* normal glomeruli*),** crescentic** (*≥50*%* cellular crescents*),** mixed** (*<50*%* normal, <50*%* crescentic, and <50*%* globally sclerotic glomeruli*), and** sclerotic** (*≥50*%* globally sclerotic glomeruli*) [19–34].

For several reasons, age might impact the validity and clinical value of histological classification in patients with ANCA-GN. Sclerotic glomeruli are often present as the result of aging, and this can confuse histological classification. Furthermore, while ESRD is the dominant outcome in younger patients, disease and/or treatment associated mortality is more frequent among elderly patients [5, 7] and might act as a competing end-point in survival analyses. In previous studies, histological classification has not been found to be useful for predicting survival among patients with ANCA-GN [20]. Additionally, the risk associated with performing a kidney biopsy increases in elderly patients due to age itself, as well as comorbid conditions and the use of concomitant medications [35]. Histological confirmation is preferred but is not strictly necessary for diagnosing ANCA-GN. Therefore, should renal biopsy be performed in very old patients is an important clinical question [36]. Evaluation of the prognostic value of the histological classification of biopsy in elderly patients with ANCA-GN is an important factor to consider when addressing this clinical question.

Data on the prognostic value of histological classification specifically in elderly patients with ANCA-GN are missing. Here, using information from the Norwegian Kidney Biopsy Registry (NKBR) and the Norwegian Renal Registry, we have analyzed the prognostic value of the histological classification in ≥70-year-old patients with ANCA-GN at the time of diagnosis. Data stratified by age of 70–74.9* versus* ≥75 years were also analyzed using both ESRD and death as end-points.

## 2. Materials and Methods

The study was approved by the Regional Committee for Medical and Health Research Ethics.

### 2.1. Identification of Study Cohort and Baseline Data

The study group was selected from the research cohort used in our previously published article “Prognostic Value of Histologic Classification of ANCA-Associated Glomerulonephritis” [20] and included patients with a biopsy-confirmed diagnosis of ANCA-GN who were ≥70 years old at the time of diagnosis. Our procedures for identifying our study cohort, the baseline data of our cohort, the histological classification of patients, definition of the period of observation, and identification of the end-points for analysis (ESRD and death) are described in detail in our previous paper, with relevant information for this study summarized as follows.

All patients were ≥70 years old at the time of diagnosis. Diagnosis was based on examination of biopsy tissue containing at least 10 glomeruli, with histological confirmation of ANCA-GN. All patients and their relevant data were identified from the NKBR between 1991 and 2012. The criteria for ANCA-GN were a pauci-immune necrotizing glomerulonephritis together with a positive ANCA titer. The following baseline data were obtained from the NKBR for analysis: sex, age, serum albumin, systolic blood pressure and diastolic blood pressure, and proteinuria. ANCA serotype was reported as analyzed by the clinician's laboratory at time of diagnosis. In the first part of the study period, ANCA was determined by the indirect immunofluorescent method; after approximately year 2000, the enzyme-linked immunosorbent assay method has been used. Estimated GFR (eGFR) was calculated from serum creatinine using the modified Modification of Diet in Renal Disease formula. Before year 2000, serum creatinine level was not standardized to isotope-dilution mass spectrometry and therefore was reduced by 5%. One of the authors (Leif Bostad), an experienced renal pathologist, classified all cases according to the histological classification schema of ANCA-GN.

### 2.2. Definition of Observation Period and Study End-Points

The observation period was from the time of biopsy to a first event of ESRD, death, or the end of the period of observation on 31 December 2012. For analysis, the observation period was stratified into the induction (≤1 year after biopsy) and the remission (>1 year after biopsy) phases. The primary end-point of this study was ESRD, defined as initiation of chronic renal replacement therapy in the form of dialysis or renal transplantation. ESRD cases were identified by linking the study cohort to the Norwegian Renal Registry. The secondary end-point of analysis was death, identified by linking the study cohort to the Population Registry of Norway. Both datasets were used to identify patients who developed ESRD or died, whichever came first, over the follow-up period of the study.

### 2.3. Statistical Analysis

Cases were classified into the focal, mixed, crescentic, and sclerotic histological types and a between-group comparison of baseline data was performed. Baseline data were subsequently compared between patients who survived and those who progressed to ESRD or died during the induction phase. The 1-year and 5-year cumulative ESRD-free survival was evaluated using Kaplan-Meier statistics, both for the entire study cohort and after stratification based on the histological classification. These analyses were repeated using the secondary end-points, namely, death and ESRD/death. Baseline data and outcomes were reanalyzed after stratification into two age groups, 70–74.9 years* versus *≥75 years at time of diagnosis. Finally, we compared the outcomes of our study cohort to outcomes previously reported in the two largest studies on ANCA vasculitis in elderly patients. For significance testing, the chi-squared test was used for categorical variables, the Mann–Whitney *U* test for continuous variables, and the log-rank test for comparisons of survival. All analyses were performed using SPSS (version 24).

## 3. Results

### 3.1. Cohort Characteristics and Outcomes

Our study cohort included 81 patients (45 women (56%) with a mean age of 77 years (standard deviation (SD), 5 years)). 47 (58%) patients were perinuclear- (P-) or myeloperoxidase- (MPO-) ANCA positive. The mean eGFR was 27 ml/min/1.73 m^2^ (SD, 23) and 38% (SD, 28%) of glomeruli presented without crescents or global sclerosis. Other relevant baseline characteristics are presented in Table 1.

The median observation period was 2.2 years (25th–75th percentiles, 0.2-5.6 years), with 279 patient years. During the short-term follow-up period (≤1 year), 15 (19%) patients required initiation of chronic renal replacement therapy and 17 (21%) patients without ESRD died.

**Figure 1:**
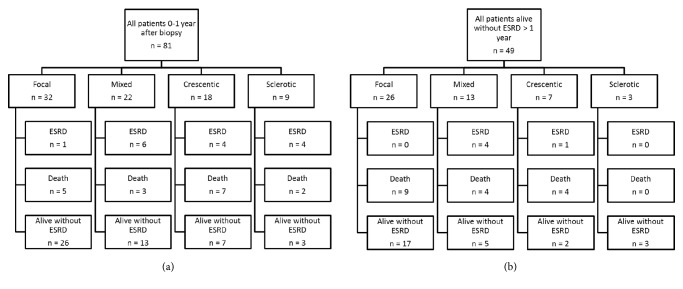
(a) Flowchart of patient outcomes at 0-1 year of observation. (b) Flowchart of patient outcomes at >1 year of observation.

49 (60%) patients survived >1 year after the diagnosis of ANCA-GN without ESRD, with 5 (6%) of these patients later progressing to ESRD. At the end-point of the study in 2012, 17 (21%) patients without ESRD had died. Causes of death were cardiovascular disease, 21%, malignancies, 12%, vasculitis/treatment associated infection, 45%, and other causes, 21%. 27 patients (33%) were alive without ESRD (Figures 1(a) and 1(b)). At the study end-point, only 4 of the 20 patients with ESRD had survived.

Baseline data stratified by histological classification are reported in Table 1. Baseline eGFR was different among the 4 groups (*p* = 0.001), with a mean eGFR of 36 ml/min/1.73 m^2^ for the focal group, 27 for the sclerotic group, 22 for the mixed group, and 15 for the crescentic group. Finally, the percentage of normal glomeruli was also different among the 4 groups, with 68% normal glomeruli in the focal group, 24% in the mixed group, 14% in the crescentic group, and 4% in the sclerotic group (p < 0.001).

### 3.2. Comparison of Survivors and Nonsurvivors

Comparison of baseline characteristics between the 49 patients who survived 1 year after ANCA-GN diagnosis without ESRD (survivors) and the 32 patients who progressed to ESRD or died in the first year after diagnosis (nonsurvivors) is reported in Table 2. Between-group differences were identified for the following baseline characteristics: percentage of P-/MPO-ANCA, 67* versus* 44% for survivors and nonsurvivors, respectively; eGFR, 33* versus 18* ml/min/1.73 m^2^, respectively; serum albumin, 32* versus* 28 gram/l, respectively; proteinuria, 1.1* versus* 1.8 gram/24 h, respectively; percentage of normal glomeruli, 49* versus* 21%, respectively; and percentage with focal (53* versus* 19%, resp.) and crescentic (14* versus* 34%, resp.) histology.

### 3.3. Cumulative Risk of ESRD, Death, and ESRD/Death Stratified for Histological Classification

**Figure 2:**
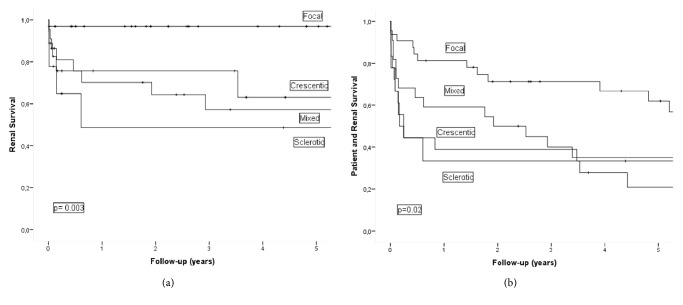
(a) Kaplan-Meier plot showing end-stage renal disease-free survival according to histological classification. (b) Kaplan-Meier plot showing patient and end-stage renal disease-free survival according to histological classification.

Cumulative survival at 1 and 5 years without ESRD among whole study cohort and after stratification according to the histological classification model is reported in Table 3(a) and Figure 2(a). The 1-year ESRD-free survival and 5-year ESRD-free survival for the whole study cohort were 80% and 73%, respectively. Survival was different among the 4 histological groups (*p* = 0.003), with 1-year survival and 5-year survival of 97% and 97%, respectively, for the focal group, 70% and 57% for the mixed group, 76% and 63% for the crescentic group, and 49% and 49% for the sclerotic group. The corresponding percentages for death at 1 year and 5 years, including death occurring after commencement of renal replacement therapy, were 73% and 50%, respectively, for the whole study group, with no difference among the 4 histological groups (*p* = 0.30; Table 3(b)): 81% and 62%, respectively, for the focal group; 73% and 39% for the mixed group; 56% and 44% for the crescentic group; and 78% and 56% for the sclerotic group. Cumulative 1-year survival and 5-year survival without ESRD/death were 61% and 42%, respectively, for the whole study group, with a significant difference between the 4 histological groups (*p* = 0.02): 81% and 62%, respectively, for the focal group; 59% and 35% for the mixed group; 39% and 21% for the crescentic group; and 33% and 33% for the sclerotic group (Table 3(c) and Figure 2(b)).

### 3.4. Comparison of Patients Aged 70–74.9* versus* ≥75 Years

**Figure 3:**
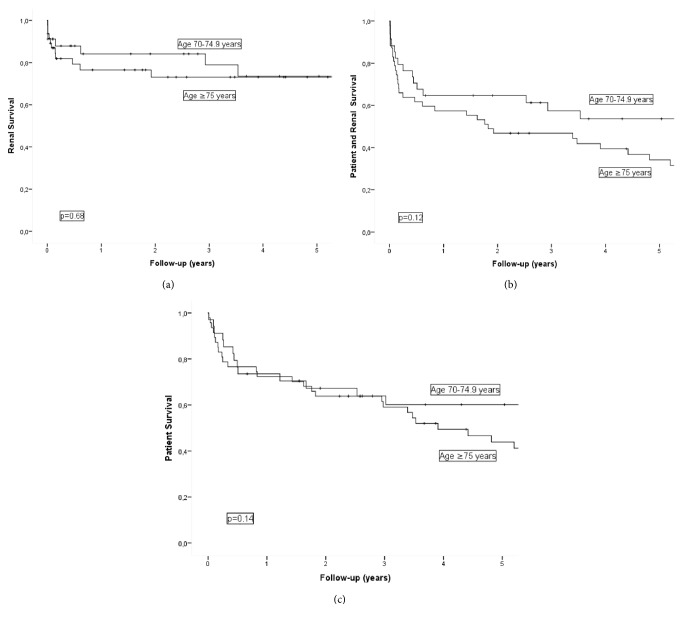
(a) Kaplan-Meier plot showing end-stage renal disease-free survival in patients aged 70–74.9 years versus ≥75 years. (b) Kaplan-Meier plot showing patient survival in patients aged 70–74.9 years versus ≥75 years. (c) Kaplan-Meier plot showing patient and end-stage renal disease-free survival in patients aged 70–74.9 years versus ≥75 years.

Comparison of baseline data according to age stratification, 70–74.9* versus* ≥75 years, is reported in Table 4. Between-group differences were identified only for mean age, 73 and 80 years, respectively (*p* < 0.001). Cumulative survival (0 to 5 years after biopsy) without ESRD, death, and ESRD/death for each age group is shown in Figures 3(a)–3(c). For the 70–74.9* versus* ≥75 years group, respectively, the cumulative ESRD-free 1-year survival was 84% and 77%, with 5-year survival of 74% and 73%. The cumulative patient survival was 74% and 27% at 1 year and 60% and 44% at 5 years, with cumulative 1-year survival without ESRD/death of 65% and 57% and 5-year survival of 54% and 34%. There was no effect of age group on the survival without ESRD or death and ESRD/death across the 4 histological groups.

### 3.5. Comparison of Outcomes Reported in International Studies

Bomback et al. reported the 1-year risk of ESRD, deaths, and the combination of ESRD and death among 61 patients, >80-year-old, with ANCA-GN [7]. ESRD and death risk were reported stratified according to the use or nonuse of immunosuppressive therapy in treated and untreated groups, respectively. The 1-year ESRD-free survival was 64% for the treated group and 27% for the untreated group. Specifically for patients >80 years old in our study group, the 1-year ESRD-free survival was 81%, compared to 54% for the treated group and 36% for the untreated group in Bomback et al.'s study. Our overall 1-year survival rate for patients >80 years old was 74%. Finally, the 1-year rate of survival without ESRD for this group of patients was 65% in our study, compared to the 30% reported by Bomback et al.

Weiner et al. reported the 1-year and 2-year ESRD free-survival and patient survival among 151 patients, >75 years old, with ANCA vasculitis from Sweden, UK, and the Czech Republic [5]. The ESRD-free survival was 75% at both the 1-year and 2-year time point of follow-up. For patients >75 years old in our study cohort, the ESRD-free survival rate was 77% at 1 year and 73% at 2 years. With regard to overall patient survival, Weiner et al. reported a rate of 72% at 1 year and 65% at 2 years, compared to 72% and 64%, respectively, in our study cohort.

## 4. Discussion

To our knowledge, this is the first study reporting the prognostic value of histological classification in elderly patients (≥70 years) with ANCA-GN, and we demonstrate that the classification predicts ESRD-free survival in this group. This finding demonstrates that, despite the possibility for sclerotic glomeruli due to aging which might affect patient classification, histological examination still has prognostic value for elderly patients. Moreover, the prognostic value of histological classification is not lost due to deaths prior to ESRD acting as a competing end-point. Finally, no fatal complications following kidney biopsy were reported.

Among our study cohort, however, histological classification was not predictive of overall survival of patients with ANCA-GN, which is slightly surprising given the high mortality rate among elderly patients having advanced kidney function impairment or receiving renal replacement therapy. As in other studies reporting outcomes in elderly patients with ANCA vasculitis, death significantly outnumbers ESRD as the end-point of analysis [5, 7]. Given this high disease and treatment associated mortality rate, it would be reassuring for clinicians to have a tissue confirmed diagnosis of ANCA-GN when treating this patient group. Thus, although tissue confirmation is not strictly necessary to diagnose ANCA-GN, our findings support the clinical value of kidney biopsy, whenever feasible, including in elderly patients [36].

Comparison of baseline characteristics stratified by histological classification demonstrated that the percentage of normal glomeruli and initial eGFR was significantly higher among patients with focal histology, which was in agreement with previous studies in all cohorts having reported the histological classification of patients with ANCA-GN.

We identified a number of factors that can influence the probability of a patient being alive without ESRD at 1-year after diagnosis, including eGFR, serum albumin, proteinuria, percentage of normal glomeruli, focal histology, and crescentic histology. These factors have been identified in previously published research and, therefore, were not surprising [4]. Interestingly, although female sex tended to be associated with a better outcome, this between-sex difference did not reach statistical significance. It is, however, surprising that P-/MPO-ANCA as compared to C-/PR3-ANCA positivity was associated with a significantly better outcome. Several previous studies including younger age groups have identified P-/MPO-ANCA positive serology as a negative prognostic marker for both progression to ESRD and patient survival [37].

Our comparison of the effects of age, stratified into 70–74.9 and ≥75 years, demonstrated no significant differences in baseline characteristics and outcomes. These findings indicate that age ≥70 years could be a reasonable cutoff for studies reporting outcomes in elderly patients with ANCA-GN.

Comparisons of outcomes for our study cohort to those from Bomback et al. and Weiner et al. demonstrated that prognosis in elderly Norwegian patients with ANCA-GN is at least comparable to that of other European countries and USA [5, 7]. Obviously clinical outcomes will depend on both the delay in diagnosis and the quality of therapy; the extent to which these factors influence outcomes, however, is uncertain.

The strengths of our study include the relatively large study cohort, its population-based nature, and identification of end-points during follow-up from high-quality national registries. Some weaknesses must also be recognized. Primarily, the treatment of individual patients is not known. We do know that cyclophosphamide and steroids were almost exclusively used for the treatment of ANCA-GN in Norway up to 2012, with exchange of cyclophosphamide for azathioprine being used for maintenance treatment after 2003 [3, 17, 20]. We do not believe that Norwegian nephrologists have performed diagnostic kidney biopsies in patients with ANCA-GN unless treatment with immunosuppression was an option; however, we cannot completely rule out the possibility that some patients were untreated. Bomback et al. and Weiner et al. reported a less favorable outcome in the relatively few patients (11 and 7 patients, resp.) not treated with immunosuppressive drugs [5, 7]. Although these findings obviously might be confounded by indication for treatment, the natural course of untreated ANCA-GN also supports the use of immunosuppression for treatment of this disease, including in elderly patients. The fact that the outcomes of patients in the present study cohort were at least in line with previous findings strongly indicates that cases included in our study group were generally treated according to established international standards. Another weakness of our study is the lack of information regarding extrarenal vasculitis activity. Such data have not been registered in the NKBR, which primarily has focused on kidney disease. In Weiner et al.'s study, the Birmingham Vasculitis Activity Score did not associate with outcomes in the multivariate analysis, indicating that the lack of such data in the present study perhaps is not a major weakness [5].

In conclusion, we have demonstrated that histological classification predicts risk of ESRD including in elderly patients (≥70 years) with ANCA-GN, and this finding supports the diagnostic value of kidney biopsy for establishing both the diagnosis and prognosis. Therefore, when feasible, a biopsy should be performed to confirm diagnosis, including in elderly patients.

## Figures and Tables

**Table 1:** Baseline characteristics for the total cohort, stratified by histological classification.

Characteristic	All, *n* = 81	Focal, *n* = 32	Crescentic, *n* = 18	Mixed, *n* = 22	Sclerotic, *n* = 9	*p* value
Female (%)	45 (56)	14 (44)	10 (56)	14 (64)	7 (78)	0.24
Age, years (SD)	77 (5)	76 (5)	77 (5)	77 (5)	76 (4)	0.79
P-/MPO-ANCA (%)	47 (58)	18 (56)	11 (61)	11 (50)	7 (78)	0.55
eGFR, ml/min/1.73 m^2^ (SD)	27 (23)	36 (24)	15 (9)	22 (19)	27 (36)	0.001
Serum albumin, gram/l (SD)	30 (6)	32 (6)	28 (4)	31 (5)	30 (5)	0.06
Systolic blood pressure (SD)	147 (23)	143 (21)	144 (27)	149 (23)	158 (24)	0.48
Diastolic blood pressure (SD)	79 (12)	80 (14)	81 (13)	77 (9)	83 (6)	0.37
Proteinuria, gram/24 h (SD)	1.4 (1.4)	1.1 (1.2)	1.2 (0.9)	1.7 (1.8)	1.4 (1.2)	0.39
Percentage of normal glomeruli (SD)	38 (28)	68 (12)	14 (16)	24 (11)	4 (6)	<0.001

SD, standard deviation.

**Table 2:** Baseline characteristics stratified for presence or absence of end-stage renal disease-free survival at 1 year.

Characteristic	Alive without ESRD, *n* = 49	ESRD/death < 1 year, *n* = 32	*p* value
Female (%)	29 (59)	16 (50)	0.42
Age (SD)	76 (5)	77 (5)	0.82
P-/MPO-ANCA (%)	33 (67)	14 (44)	0.64
eGFR (SD)	33 (22)	18 (22)	<0.001
Serum albumin (SD)	32 (6)	28 (5)	<0.001
Systolic blood pressure (SD)	146 (22)	147 (25)	0.89
Diastolic blood pressure (SD)	78 (12)	81 (11)	0.55
Proteinuria (SD)	1.1 (1.1)	1.8 (1.6)	0.02
Percentage of normal glomeruli (SD)	49 (26)	21 (23)	<0.001
Focal (%)	26 (53)	6 (19)	0.002
Mixed (%)	13 (27)	9 (28)	0.88
Crescentic (%)	7 (14)	11 (34)	0.03
Sclerotic (%)	3 (6)	6 (19)	0.08

SD, standard deviation; eGFR, estimated glomerular filtration rate.

**Table:** (a) End-stage renal disease-free survival at 1 year and 5 years for the total cohort, stratified by histological classification

Characteristic	N	ESRD	1 year	5 years	*p* value
All	81	20	80 %	73 %	
Focal	32	1	97 %	97 %	
Mixed	22	10	70 %	57 %	
Crescentic	18	5	76 %	63 %	*p* = 0.003
Sclerotic	9	4	49 %	49 %	

**Table:** (b) Patient survival at 1 year and 5 years for the total cohort, stratified by histological classification

Characteristic	N	Death	1 year	5 years	*p* value
All	81	50	73 %	50 %	
Focal	32	15	81 %	62 %	
Mixed	22	16	73 %	39 %	
Crescentic	18	14	56 %	44 %	*p* = 0.30
Sclerotic	9	5	78 %	56 %	

**Table:** (c) Patient and end-stage renal disease-free survival at 1 year and 5 years of follow-up in total cohort, stratified according to histological classification

Characteristic	N	ESRD/death	1 year	5 years	*p* value
All	81	54	61 %	42 %	
Focal	32	15	81 %	62 %	
Mixed	22	71	59 %	35 %	
Crescentic	18	16	39 %	21 %	*p* = 0.02
Sclerotic	9	6	33 %	33 %	

**Table 4:** Baseline characteristics stratified by age, 70–74.9 years and ≥75 years, at time of inclusion.

Characteristic	Age 70–74.9 years, *n* = 34	Age ≥75 years, *n* = 47	*p* value
Female (%)	18 (53)	27 (57)	0.68
Age, years (SD)	72 (1)	80 (4)	<0.001
P-/MPO-ANCA (%)	20 (59)	27 (57)	0.90
eGFR, ml/min/1.73 m^2^ (SD)	30 (25)	25 (22)	0.15
Serum albumin, gram/liter (SD)	31 (7)	30 (5)	0.46
Systolic blood pressure (SD)	145 (22)	148 (24)	0.52
Diastolic blood pressure (SD)	81 (11)	78 (13)	0.43
Proteinuria, gram/24 hours (SD)	1.5 (1.2)	1.3 (1.5)	0.06
Percentage of normal glomeruli (SD)	44 (26)	34 (29)	0.13
Focal histology (%)	17 (50)	15 (32)	0.10
Mixed histology (%)	8 (24)	14 (30)	0.53
Crescentic histology (%)	6 (18)	12 (26)	0.40
Sclerotic histology (%)	3 (9)	6 (13)	0.58

SD, standard deviation; eGFR, estimated glomerular filtration rate.
